# Molecular Links between Central Obesity and Breast Cancer

**DOI:** 10.3390/ijms20215364

**Published:** 2019-10-28

**Authors:** Alina-Andreea Zimta, Adrian Bogdan Tigu, Maximilian Muntean, Diana Cenariu, Ondrej Slaby, Ioana Berindan-Neagoe

**Affiliations:** 1 MEDFUTURE-Research Center for Advanced Medicine, University of Medicine, and Pharmacy Iuliu-Hatieganu, 23 Marinescu Street, 400337 Cluj-Napoca, Romania; andreea.zimta@umfcluj.ro (A.-A.Z.); adrianbogdantigu@gmail.com (A.B.T.); diacenariu@gmail.com (D.C.); 2 Babeș-Bolyai University, Faculty of Biology, and Geology, 42 Republicii Street, 400015 Cluj-Napoca, Romania; 3 Department of Plastic Surgery, University of Medicine and Pharmacy “Iuliu Hatieganu”, 400337 Cluj-Napoca, Romania; maximilian.muntean@gmail.com; 4 Central European Institute of Technology, Masaryk University, 62100 Brno, Czech Republic; on.slaby@gmail.com; 5 Masaryk Memorial Cancer Institute, Department of Comprehensive Cancer Care, 60200 Brno, Czech Republic; 6 Research Center for Functional Genomics, Biomedicine and Translational Medicine, “Iuliu Hatieganu” University of Medicine, and Pharmacy, 23 Marinescu Street, 400337 Cluj-Napoca, Romania; 7 Department of Functional Genomics, and Experimental Pathology, The Oncology Institute “Prof. Dr. Ion Chiricuta”, Republicii 34th street, 400015 Cluj-Napoca, Romania

**Keywords:** breast cancer, abdominal fat, obesity, menopause, hormone dependency, leptin, adiponectin, miRNA, exosomes

## Abstract

Worldwide, breast cancer (BC) is the most common malignancy in women, in regard to incidence and mortality. In recent years, the negative role of obesity during BC development and progression has been made abundantly clear in several studies. However, the distribution of body fat may be more important to analyze than the overall body weight. In our review of literature, we reported some key findings regarding the role of obesity in BC development, but focused more on central adiposity. Firstly, the adipose microenvironment in obese people bears many similarities with the tumor microenvironment, in respect to associated cellular composition, chronic low-grade inflammation, and high ratio of reactive oxygen species to antioxidants. Secondly, the adipose tissue functions as an endocrine organ, which in obese people produces a high level of tumor-promoting hormones, such as leptin and estrogen, and a low level of the tumor suppressor hormone, adiponectin. As follows, in BC this leads to the activation of oncogenic signaling pathways: NFκB, JAK, STAT3, AKT. Moreover, overall obesity, but especially central obesity, promotes a systemic and local low grade chronic inflammation that further stimulates the increase of tumor-promoting oxidative stress. Lastly, there is a constant exchange of information between BC cells and adipocytes, mediated especially by extracellular vesicles, and which changes the transcription profile of both cell types to an oncogenic one with the help of regulatory non-coding RNAs.

## 1. Introduction

Breast Cancer (BC) is the most common type of cancer among women. In recent years, the role of environmental exposure in this malignancy development is becoming increasingly recognized [1]. Despite the latest research in the targeted breast cancer drug delivery of s [2,3] and the involvement of non-coding RNAs in combined therapy [4], this malignancy still poses a great threat to women’s health, which is why a better understanding of its prevention will have a major impact on the general population of women.

There are three surface receptors commonly used to characterize BC. These are: the estrogen receptor (ER), the progesterone receptor (PR), and the human epidermal growth factor receptor 2 (HER2). According to their presence or absence, BC is divided into: ER^+/−^, PR^+/−^, and HER2^+/−^. The triple negative BC (TNBC), ER-/PR-/HER2-, has the highest mortality rate due to its lack of an efficient therapeutic target [5,6]. Apart from the definitive role that general adiposity plays in BC development, it seems that fat distribution is also important [7]. Multiple studies have proved the association between BC and central obesity in women; however, these conclusions are many times dependent on the menopausal status.

A ten-year prospective study on Taiwanese women concluded that a high BMI has the opposite effect over BC incidence in pre-menopausal versus post-menopausal women. Before menopause, there is no correlation between BC and obesity; while in post-menopausal women [8], with every 5-unit increase in BMI there is a 33% increase in the risk of developing ER+/PR+ BC. In pre-menopausal women, high BMI translates into a decrease in BC risk by 10% [9]. The post-menopausal women have a higher risk of developing ER+/PR+ BC, because the excess in fat tissue stimulates sex hormone synthesis, and exposure of breast tissue, at a time point when the body is no longer adapted for reproduction [8,10,11]. However, after BMI adjustment, central obesity is correlated with BC only in pre-menopausal women. Central obesity has no connection with BC in post-menopausal women [12]. As follows, pre-menopausal women with increased central adiposity have a higher density of breast tissue [13], and an increased risk of developing triple-negative BC [14,15,16]. The C57BL/6 mice were induced in menopause by ovariectomy, subsequently given high-fat food, and injected with BC cells. The obese mice had a higher tumor size in comparison with the non-obese mice. The post-menopausal mice also had a higher visceral adipose tissue (VAT) to the total body mass ratio [17].

Being overweight is not sufficient for the development of BC, which may be associated with inherited genetic predisposition, such as mutations in the leptin receptor (*LEP* rs7799039 AA or LEPRrs1137100 GG) [18]. A combination of mutations in long non-coding RNA(LINC00460 rs17254590) and Muskelin 1 gene (*MKLN1* rs117911989), associates with a higher BMI and increased risk of developing BC [19]. Another study concluded that there is no link between diabetes and BC [20], however Metformin is an example of an anti-diabetic drug, which has been successfully repositioned to lower the risk of BC in obese women [21,22].

The link between obesity and BC has been extensively studied. A recent review reported 12 clinical trials which evaluate the impact of obesity over BC treatment efficiency, and how healthier lifestyle choices may impact disease progression. They also analyzed the mechanism behind these observations based on stimulation of inflammation, hypoxia, and hormonal unbalance [23].

The role of central obesity in developing BC is supported by strong epidemiological and clinical evidence, however, little is known about the mechanisms behind these observations. This review proposes a view of the association between central obesity and BC, from the point of view of signaling pathways activated/repressed by estrogen, leptin, and adiponectin, changes in the adipose tissue microenvironment and its effect over systemic inflammation, and changes in the expression level of multiple protein-coding genes and microRNAs.

## 2. Tumor Microenvironment vs. Adipose Tissue Microenvironment and Their Role in Tumor-Promoting Low-Grade Inflammation

The tumor tissue is highly heterogeneous, being composed of several cell types which are reprogrammed to sustain tumor growth and spread. Tumor immune cells belong to both the innate immunity and adaptive immunity. The most abundant immune cells are the macrophages. The majority of macrophages are the tumor-associated macrophages (TAMS). They have an anti-inflammatory phenotype and are the result of oncological transformation from normal macrophages which no longer possess the capacity of detecting and attacking cancer cells [24]. In the tumor, the cytotoxic CD8+ cytotoxic T cells, and CD4+ T helper cells have a reduced number compared to normal tissue [25]. Moreover, both types of T cells are unresponsive due to their state of anergy [26]. This state of immune tolerance is further accentuated by the presence of a high number of regulatory T cells (T_regs_), type 2 T helper cells (Th2) [27], and regulatory B cells (B_regs_). This population has the important function of down-regulating the immune response through the secretion of anti-inflammatory cytokines, especially of IL-10 [28]. The adipose tissue is divided into two main categories: the brown adipose tissue and the white adipose tissue. The brown adipose tissue is abundant during in utero life, followed by a regression during adulthood, when it is responsible for nonshivering thermogenesis, such as in the case of febrile state [29]. The white adipose tissue (WAT) is responsible for long-term storage of energy, in the form of triglycerides [30], and it is divided into two types, depending on the body distribution: the subcutaneous fat and the visceral fat. Subcutaneous fat is distributed all over the body under the dermis, while visceral fat is located in the abdominal cavity, in the omentum and mesenteric area [31]. The adipose tissue microenvironment is heterogeneous, and it contains a specific pattern of cells depending on the body localization. Visceral adipose tissue (VAT) contains more pro-inflammatory immune cells, a smaller population of preadipocytes, and a larger number of differentiated adipocytes [31], fibroblasts, macrophages [32], and a special subset of OX40-expressing Tregs [33]. This subpopulation is known to down-regulate the anti-inflammatory phenotype of the local milieu [34].

The white adipose tissue, both subcutaneous and visceral, is characterized by an increased number of mast cells which are pro-inflammatory immune cells and accumulate especially in the VAT of obese people [35,36,37,38]. The mast cells are involved in tissue repair by secreting proinflammatory cytokines, chemokines, and growth factors [37,38]. Moreover, in obese VAT is a higher abundance of leptin-sufficient mast cells [36]. 

The natural killer (NK) cells are in smaller number in the obese adipose tissue, compared to normal weight adipose tissue. The exposure of NK cells to a higher concentration of leptin decreases their capacity of IFN-γ production [39]. Moreover, in esophageal cancer it was proven that the visceral adipose tissue induces NK cell apoptosis [40]. The natural killer cells from the adipose tissue have a special, cancer-promoting phenotype. They underexpress the NKp30, and NKp44, and are less effective in eliminating tumoral tissue. There is no difference regarding the loss of this capacity in obese versus lean people [41]. However, other authors stated that the loss of function of NK cells is more common in obese people [42]. The visceral adipose tissue has a higher population of NK cells [43], while in BC this population is reduced [44]. In the case of obesity, the visceral adipose tissue has a higher population of M1 CD40+ macrophages compared to the M2 macrophages. With a pro-inflammatory phenotype, the M1 macrophages secrete pro-inflammatory cytokines [45,46,47], and express specific surface molecules such as: CD16 and CD36 [43,48]. On the other hand, the M2 macrophages have an anti-inflammatory role and are activated by IL-13 or IL-4 [45,46,47]. There is an increased population of macrophages in the adipose tissue of obese people. The M1 macrophage secretes pro-inflammatory cytokines, called adipokines [32], especially IL-6, and the C-reactive protein (CRP) which leads to a raise in systemic inflammation [49]. Chronic inflammation and an increase in the macrophage-secreted tumor necrosis factor (TNF)-α, interleukin (IL)-6, and IL-1β, have been linked to a breakthrough in BC onset [50].

One of the most important mechanisms behind the observed association between central obesity and breast cancer is the systemic release of pro-inflammatory cytokines, which causes insulin resistance, and alterations in the insulin-initiated signaling pathways, which are linked to BC development [51]. VAT has a small number of macrophages in lean people [52], but this number greatly increases in the VAT of obese people [53]. A more complete list of pro-inflammatory cytokines, and their role in BC development is found in Table 1.

Chronic inflammation has a tumor-promoting activity, due to the release of reactive oxygen species (ROS) by inflammation-induced necrosis and the activity of polymorphonuclear cells. In a normal cell, the activity of various transcription factors (TFs) increases as a response to inflammation. These TFs are: NFκB, STAT3, NRF2, AP1, HIF1-α, and PPARα. This overactivity of DNA synthesis and transcription leads to DNA/RNA mutations [69]. Constant, prolonged, and moderated levels of ROS contribute to the induction of BC through the activation of AKT tyrosine kinase [70]. Moreover, the ROS association with the pro-inflammatory cytokine interleukin 6 (IL-6) is positively correlated with the lymph node involvement in ER+ BC, and distant metastasis formation in the case of ER- BC [71]. Obesity-associated inflammation changes the splicing preference of methyl-CpG-binding domain protein 2 (MBD2), thus resulting in a greater degree of MBD2_variant2, which in turn promotes the maintenance of cancer stem cells in triple negative BC [72]. In a study, the THP1 cells, a macrophage model, were activated by lipopolysaccharides and their culture media were placed on top of visceral mature adipocytes. These adipocytes were shown to increase their IL-6, IL-8, chemokine (C-X-C motif) ligand 1 (CXCL1), and C-C motif chemokine ligand 2 (CCL2) mRNA expression, and protein levels. This led to de novo secretion of intercellular adhesion molecule 1 (ICAM1), IL-1β, interferon gamma-induced protein 10 (IP-10), macrophage inflammatory protein 1alpha (MIP-1a), macrophage inflammatory protein 1 beta (MIP-1β), vascular endothelial growth factor (VEGF), and tumor necrosis factor alpha (TNFα) [73]. The SphK1/S1P/S1PR1 axis promotes the production of pro-inflammatory cytokines, macrophage infiltration, and BC tumor progression [74].

A recent study evaluated the interaction between the subcutaneous adipose-derived stem cell (ATC), visceral adipose stem cells and double positive BC cells. It was concluded that the co-culture between the two cell types leads to the increased proliferation and invasion capacity of malignant cells. This is reasoned by the fact that the VAT stem cells establish a tighter interaction with the BC cells. In addition, the pro-inflammatory cytokines IL-6 and IL-8 are secreted in greater quantity [75].

The inflammasome is a multiprotein complex responsible for the induction of inflammation through the production of IL-1β and IL-18 [76]. The NLRP3 inflammasome is overexpressed in the visceral adipose tissue compared to the subcutaneous tissue, and it triggers a greater secretion of the IL-1β, IL-18, IL-6, IL-8, and caspase-1. The majority of VAT secreted cytokines are produced by the CD8+ T cells, present in a greater number in the VAT [52]. By comparing BC tumor formation in caspase-1 knock-out (KO) mice, and NLRP3 KO C57BL/6 mice with the wild-type mice, it was proven that the NLRP3 inflammasome is the most common type of inflammasome formed in breast malignant tissue [54]. The inflammatory pattern of obese BC patients revealed that IL-1β is a mediator of obesity-associated BC. By using *Nlrp3*^−/−^, *Nlrc4*^−/−^, *Casp1/11*^−/−^, and WT obesity-prone C57BL/6 mice, another study concluded that the NLRC4 inflammasome, associated often with obesity, has a more important role in BC progression than the NLRP3 inflammasome. This inflammasome, by producing IL-18, recruits myeloid derived cells, and by a local increase in IL-1β, stimulates VEGFα secretion in the adipose tissue [77].

## 3. The Hormones, Their Signaling Pathways, Central Adiposity, and Breast Cancer

The adipose tissue can be regarded as an endocrine organ. This function is altered in the case of obesity. The VAT resistin level is decreased in the case of post-menopausal women by 0.69-fold [78]. An increased level of resistin and visfatin was suggested to be associated with a higher number of macrophages in the VAT of obese people, since these two hormones seem to sustain inflammation [79]. The three most important hormones are leptin, adiponectin and resistin. The adiponectin level is lowered, while the leptin and resistin values are increased [80].

### 3.1. Leptin

Leptin has an increased level in obese people and it is positively correlated with overall weight [81]. This hormone causes a switch of macrophages towards M2 phenotype, in a VAT microenvironment [36]. A meta-analysis from 2013 found that a high circulating leptin level is associated with a greater risk of BC [82]. Leptin stimulates the secretion of the pro-inflammatory cytokines, IL-6, IL-1, IL-17, TNF-α, and TGF-β-, thus providing a greater chance of BC progression [83]. In ERα+ BC, leptin promotes cell viability and migration through the JAK/AKT/STAT-pathway [84]. Leptin binds to the ERα and induces cell cycle progression by stimulating the expression of cyclin D [85].This hormone promotes cell proliferation, and in vitro, it inhibits the expression of adiponectin receptors (AdipoR1 and AdipoR2) [86]. In the breast tumor microenvironment, leptin induces VEGF production by cancer associated fibroblasts (CAF), immune cells, and normal adjacent epithelial cells [87,88].

### 3.2. Adiponectin

**Figure 1:**
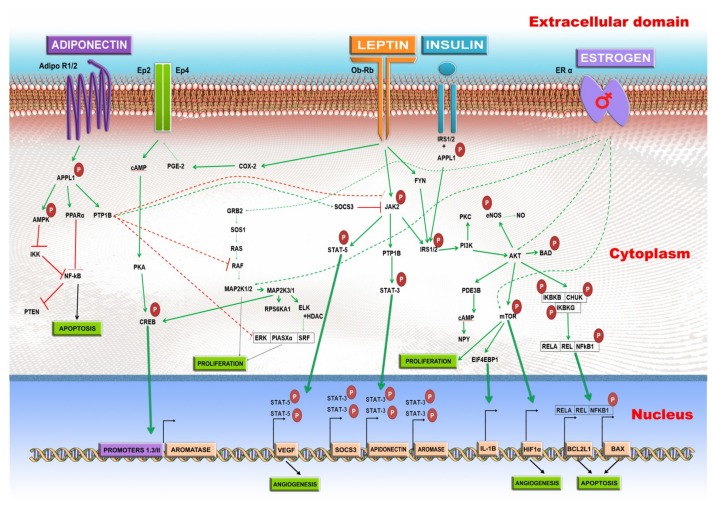
The pathways activated by several dysregulated hormones in central obesity. The green arrow stands for activation and the red line for repression. Leptin binds to the Ob-Rb receptor, and activates several signaling pathways. The JAK2-STAT3 pathway is activated; which will result in the stimulated transcription of the *SOCS3*, and ultimately *AROMASE (CYP19A)* genes. The JAK-STAT5 pathway results in the increased expression of vascular endothelial growth factor (VEGF), which leads to angiogenesis. The IRS1/2-PI3K-AKT is also stimulated by Ob-Rb receptor which increases the NO level, and activates BCL2 associated agonist of cell death - *BAD*. The IRS1/2-PI3K-AKT stimulation also leads to the activation of mTOR which increases the proliferation, and stimulates the expression of HIF-1α, respectively, it initiates the angiogenesis process. The AKT interaction with the IKBKB/CHUK/IKBKG complex is followed by the activation of RELA/REL/NFκB1 which will lead to the increased expression of BCL2 Like 1- *BCL2L1*, and BCL2-associated X, apoptosis regulator-*BAX,* two genes which form a complex involved in apoptosis. The AKT activation phosphorylates the mTOR pathway, leading to the stimulated transcription of HIF1α, IL-1β, and increased proliferation. The leptin – Ob-Rb interaction causes the activation of COX-2 – PGE-2, and the prostaglandin and in EP2/4 receptor. This is followed by the cAMP-PKA-CREB activation. CREB will bind to the Promoter 1.3/II of aromatase and it will stimulate the *AROMASE (CYP19A)* gene transcription. The adiponectin interaction with one of its two receptors, AdipoR1 or AdipoR2 leads to the adaptor protein, phosphotyrosine, interacting with PH domain and leucine zipper 1 APPL1 phosphorylation, which will activate protein tyrosine phosphatase non-receptor Type 1-PTP1B. The PTP1B represses Janus kinase-JAK by direct interaction or through the activation of suppressor of cytokine signaling 3 - SOCS3. The tyrosine-protein phosphatase non-receptor type 1, also known as protein-tyrosine phosphatase 1B (*PTP1B*), also leads to the repression of RAF, and the ERK/PIASX alpha/SRF complex, thus inhibiting proliferation. The APPL1-PPARα or APPL1/AMPK-IKK causes the NFκB inhibition, leading to apoptosis. The overstimulation of the ER leads to the activation of GRB2, AKT, and mTOR.

In post-menopausal women, adiponectin is underexpressed in breast tumor tissue [89], while the serum adiponectin is inversely correlated with BC only in the case of Asian women, not in Caucasian [90]. Mutations in the adiponectin gene (*ADIPOQ*) affect the occurrence of ductal infiltrating breast cancer (DIBC). The rs2241766, T > G mutation offers protection against DIBC development, while rs1501299, G > T mutation constitutes a risk factor [91]. Adiponectin causes death of BC cells due to an overstimulation of autophagy, resulting in the cellular depletion of ATP, through the induction of Unc-51-like autophagy activating kinase (ULK1/2), due to the stimulation of the MAP1LC3B-II/LC3B-II pathway, while the anti-autophagy axis SQSTM1/p62 is inhibited [92]. Estrogen has an opposite effect during the activation of the adiponectin-mediated signaling pathway. In ER+ BC cells, the activation of estrogen receptor 1 (ER1) leads to the entrapment of liver kinase B1 (LKB1), which is no longer capable of interacting with the protein kinase AMP-activated catalytic subunit alpha 1(AMPK); this being an essential signal transducer of adiponectin [93]. A detailed image over the signaling pathways activated by obesity-induced leptin, estrogen overproduction, and inhibition of adiponectin is provided in Figure 1 [94,95,96,97,98,99,100,101].

### 3.3. Estrogen

**Figure 2:**
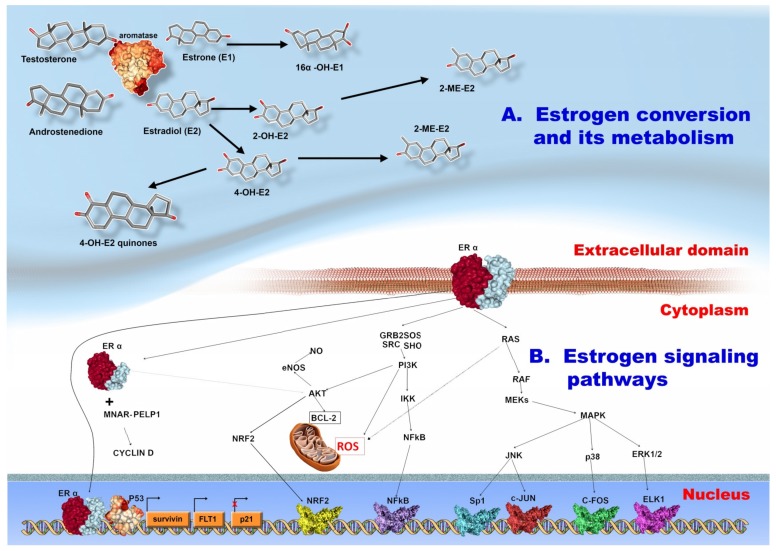
(**A**) Estrogen biogenesis, and its metabolism. The male hormones, androstenedione, and testosterone are converted by aromatase to estrone (E1) and estradiol (E2). Estrone is converted to 16α-hidroxy-estrone. The estradiol is converted to 2-hidroxy-estradiol (2-OH-E2) or to 4-hydroxy-estradiol (4-OH-E2). The 2-OH-E2 can be further metabolized to 2-methyl-estradiol. The 4-OH-E3 is also converted to 4-methyl-estradiol (4-ME-E2). The 4-OH-E2 can also interact with quinone, giving rise to the 4-hidroxy-quinones. (**B**) The signaling pathways initiated by estrogen interaction with its receptor. The ERα interaction with estrogen leads to the activation of several pathways. The RAS-RAF-MEK-MAPK pathway activates: JNK, p38, and ERK1/2 pathways. The JNK activates the Sp1, and c-JUN transcription factors. P38 leads to the activation of c-FOS and ERK1/2 to activation of ELK1. The estrogen receptor also interacts with GRB2/SOS/SRC/SHO, leading to the activation of PI3K-IKK, and the NFκB transcription factor. The PI3K activates AKT leading to the activation of the anti-apoptotic factor BCL-2, and the production of nitric oxide. The ERα can translocate to the cytoplasm or to the nucleus. In the cytoplasm, it interacts with MNAR-PELP1, and stimulates the activation of cyclin D. In the nucleus, ERα interacts with p53 and the DNA, leading to the up-regulation of *survivin* gene, Fms related tyrosine kinase 1-*FLT1*, and the down-regulation of *p21*. The 3D structure of proteins was taken from the RCSB-PDB database (https://www.rcsb.org/).

In addition to its singular effect, leptin increases the risk of BC development through its stimulation of estrogen synthesis [102], due to activation of aromatase, an enzyme responsible for the conversion of androgens into estradiol (Figure 2A) [103]. It is important to point out that local activity of aromatase alone is sufficient to maintain BC formation, independent on the systemic level of estrogen [104].

Estrogen could be the missing link in regard to the hormonal unbalance between lean and obese women. After menopause, the ovaries stop producing estrogen and the adipose tissue is the main systemic provider of estrogen. A higher degree of adipose tissue means a higher systemic production of estrogen, and the post-menopausal exposure to this sex hormone leads to BC installment [105,106]. Some researchers stated that the increased burden of converting androgen precursors in estradiol might be a cause of obesity-related BC in post-menopausal women [107]. For instance, a study done in 1991 wanted to counteract the menopause effects by giving estrogen replacement therapy to post-menopausal women, however, the treatment had a detrimental effect on women’s health, causing among others, a higher incidence of BC [108]. Continuous rise in oxidative stress, as well as estradiol (E2) stimulation, leads to NRF2 accumulation by means of P13K–AKT signaling pathway activation. Gorrini C. et al. were able to demonstrate the interplay between P13K signaling, and the NRF2 antioxidant involvement in *BRCA1-*mediated tumorigenesis [109].

There is also a hormonal equilibrium regulation between visceral versus subcutaneous adiposity. Estrogen is an important indicator of WAT distribution. In male mice fed with a high caloric diet there was a propensity to accumulate WAT in the visceral area, while in female mice, an equal distribution was observed between subcutaneous WAT and visceral WAT [110]. Estrogen secretion is related to the aromatase overactivation in the breast adipose tissue [111,112] thus, high concentrations of androsterone and testosterone are still dangerous, because the aromatase is able to convert them into estrogen, estrol, and estradiol [113]. Further details are found in Figure 2A.

Some in vivo studies demonstrated that estrogen, and catechol metabolites promote kidneys, liver, uterus and mammary gland carcinogenesis. Firstly, the estrogen carcinogenesis is based on its specific binding to a receptor and the consequent activation of tumor-promoting genes. [113,114,115]. The membrane ER is associated with a G protein. The activation of this G protein increases the cAMP, and thus the epidermal growth factor receptor (EGFR) can bind to the epidermal growth factor (EGF), which initializes a signaling chain which will activate mitogen activated protein kinase (MAPK), and phosphatidyl-inositol 3 kinase (PIP3K) [113]. Further details are illustrated in Figure 2B.

Secondly, the estrogen metabolites have different degrees of carcinogenic effect. The estrogen metabolites do not possess equal levels of carcinogenicity [116]. The tumor-promoting effects of reactive catechol estrogen quinones are based on their ability to create DNA adducts [117]. The 2-hydroxyestrone was named in an earlier review as “the good” estrogen [118], because it has anti-estrogenic activity. However, a meta-analysis concluded that the circulating estrogen metabolites were insufficiently studied in order to consider them as predictive biomarkers for BC [119]. The estrogen-receptor independent pathogenic mechanisms in BC are linked to estrogen metabolites, such as estradiol and 4-hydroxiestradiol [120].

## 4. Exosomes, MicroRNAs, and Their Possible Role in the Interplay between Central Adiposity, and BC

MicroRNA-mediated communication between different populations of the tumor microenvironment have a profound role in maintaining its homeostasis, and are important communicators between different cell types of the tumor milieu [121]. The visceral adipocyte-derived exosomes from obese people have the tumor suppressors miR-148b and miR-4269 down-regulated, while the oncomiR, miR-23b is up-regulated [122].

miR-148b functions as a tumor suppressor miRNA in BC and it down-regulates post-transcriptionally the *DNMT3b* gene [123], whose overexpression is associated with the DNA hypermethylation. This process contributes to the malignant transformation and the maintenance of the cancerous phenotype in BC [124]. MiR-148b underexpression is considered as a significant mediator of aggressive forms of BC [125]. MiR-148b has an antagonistic role with the oncomiR-214 in promoting cancer cell migration in vitro, and in vivo by modulating the expression level of the adhesion molecules integrin alpha 5 (ITGA5), and activated leukocyte cell adhesion molecule (ALCAM) [126]. The lncRNA CCAT1 is associated with chemoresistance and it seems to exert its action by down-regulating the expression level of miR-148b [127]. However, miR-148b shows an opposite level in tumor tissue versus systemic circulation of BC patients. The circulating plasma level of miR-148b is up-regulated in BC patients versus healthy controls [128,129,130].

The information regarding miR-4269 involvement in BC is scarce. A study analyzing the intra-tumoral differential distribution of microRNAs showed that in Luminal B Ki67+ area, miR-4269 has a greater expression level in the edges of the tumor compared to the central area, but at the same time, it showed that miR-4269 has a low expression level in tumor tissue versus normal tissue [131]. MiR-23 is generally considered a tumor promoter, being detected in high levels in the serum of BC patients [132]. Some agents, such as anacardic acid or sulforaphane are considered to be effective oncological treatment options, because they down-regulate miR-23b [133,134]. This microRNA does not interfere in cell proliferation or apoptosis, but it is involved in the invasion and migration process of BC malignant cells. In the MCF-7 or MDA-MD-231 cells, the overexpression of miR-23b is associated with decreased metastasis potential, cell motility and gain of epithelial phenotype. The microRNA interacts with the P21(*RAC1*) activated kinase 2 (*PAK2)* mRNA [135]. However, a study demonstrated that miR-23b is a part of the miR-23b/27b/24 cluster of synchronous microRNAs that, in BC, inhibits local invasion, but stimulates lung metastasis [136].

In mice susceptible to polygenic diabetes, the diet-induced adiposity revealed that miR-148b is decreased in the visceral adipose tissue, whereas the microRNAs from miR-200 family: miR-200a, miR-200b, miR-200c, miR-141, and miR-429 are up-regulated [137]. miR-200 family has tumor suppressor functions in BC [138]. miR-200a is a tumor suppressor that inhibits cell migration in triple negative BC by regulating the E-cadherin and oncogene EPH receptor A2 (EPHA2) [139]. The miR-200a up-regulation is associated with BC cell proliferation, through its interaction with transcription factor A, mitochondrial—*TFAM* gene. *TFAM* completes with P73 antisense RNA 1T -TP73-AS1 for the binding of miR-200a [140]. The miR-141 is also a tumor suppressor miRNA, involved in the repression of BC cell proliferation, invasion and migration [141,142]. MiR-429, by targeting zinc finger E-box-binding homeobox 1 (ZEB1) and CRK-like proto-oncogene, adaptor protein (CRKL) has also an anti-invasion activity in BC [143].

The main microRNA found in the exosomes from adipose tissue macrophages (ATM) is miR-155, a microRNA involved in insulin resistance. These particular types of exosomes are mainly secreted by the ATMs from obese adipose tissue with a higher population of necrotic cells [144]. miR-155 plays an oncogenic role in BC. It regulates many genes involved in invasion (SMAD family member 1—*SMAD1*, SMAD family member 5—*SMAD5*), apoptosis (caspase 3—*CASP3*), fas associated via death domain—*FADD*, receptor interacting protein—*RIP1*, interleukin 1 receptor associated kinase—*IRAK*, protein kinase A–*PKA*, apoptotic peptidase activating factor 1—*APAF-1*, forkhead box O3—*FOXO3A*, B-cell CLL/lymphoma 2—*BCL-2*, proliferation (suppressor of cytokine signaling 1—*SOCS1*, SMAD family member 2—*SMAD2*, angiogenesis (hypoxia inducible factor 1 subunit alpha—*HIF-1*), and differentiation (Spi-1 proto-oncogene—*SPI1*, macrophage colony-stimulating factor receptor—*MCSFR*) [145]. miR-155 interaction with tumor protein P53 inducible nuclear protein 1—*TP53INP1*, a tumor suppressor gene, leads to increased cell proliferation in BC [146].

In the interplay between adipose tissue and breast cancer tissue, the non-coding RNAs are often modulated by other environmental factors that can either have a detrimental or beneficial role. For instance, epigallocatechin gallate, curcumin and genistein constitute three phytochemicals, that by changing the expression level of microRNAs [147,148] could also partially prevent the effects of obesity over breast cancer development (Table 2).

The main component through which breast cancer cells are modulated to change their behavior as a response to obesity, is related to the long-distance communication between the adipose cells found in VAT and the cancer cells [153]. Moreover, Ji C. and Guo X. launched the idea that miRNAs generate from adipose tissue delivery through exosomes may function as an endocrine or paracrine means of intracellular communication between the adipose tissue and other tissue types, and that miRNAs may function as biomarkers [154]. The communication via non-coding RNAs can be with necked miRNAs or with miRNAs found in the extracellular vesicles, like exosomes [155].

The proof of this communication came from the exogenously induced overexpression of miR-140 in preadipocytes, which in turn secrete exosomes with high levels of miR-140. When these exosomes are internalized in MCF10DCIS (ductal carcinoma in situ) cells they inhibit the expression of SOX2 at both mRNA and protein level, and the expression of SOX9 only at the protein level. As a result, the breast malignant cells show a decreased population number of cancer stem cells and less aggressiveness in regard to migration potential. If the MCF10DCIS are treated with normal exosomes from pre-adipocyte cells, the reverse situation happens [156].

The mesenchymal stem cells (MSCs) derived from adipose tissue after tumescent liposuction is another source of exosome-derived increased aggressiveness of breast cancer cells. The MSCs originate exosomes when put on top of the double-positive breast cancer cells, MCF-7, and lead to the increased invasion capacity of MCF-7 cells and the most significant activation of the Wnt signaling pathway, as proven by the overexpression of β-catenin at the mRNA and protein level. The Wnt target genes, Axin2, and Dickkopf-related protein 1 (Dkk1) also showed increased expression level after exosome treatment [157].

The exosomes from MSC transformed adipocytes, when added to the media of MCF7 cells, changed the transcriptomic landscape of the MCF7 cells. The BC cells’ overexpressed genes involved in cell cycle progression (cyclin D1, cyclin D3), cell proliferation (proto-oncogene c-Met, MYC proto-oncogene, BHLH transcription factor), migration (transforming growth factor-beta 1, matrix metallopeptidase 9) and angiogenesis (vascular endothelial growth factor A, hypoxia- inducible factor 1 subunit alpha) [158].

The adipose tissue, especially the brown adipose tissue can repress the expression of mRNAs found at greater distance [159].

The breast cancer cells also release exosomes that alter the phenotype of adipocytes to brown adipose cells. The BC exosomes are loaded with miR-155, which represses the expression of PPARγ and ERK1/2, while increasing the expression of p38. This change reprograms the metabolism of adipocytes that produces more high-energy metabolites such as pyruvate, lactate and free fatty acids. These metabolites sustain the enhancement of invasion capacity in BC malignant cells [160].

The difference between obese and normal weight women is seen by the molecular profile present in the tumor. The difference in expression of miR-10b, between healthy tissue and tumor tissue, is lower in the case of women with normal BMI than in the case of obese women. This microRNA was down-regulated in the tumor tissue of obese women, and its targets MAPRE1, PIEZO1, SRSF1 and TP53 were up-regulated [161].

The transformation of cancer-associated adipocytes (CAA) that sustain the low-grade inflammatory milieu in the tumor tissue is mediated by changes in the miRNA profiles. For instance, the CAA from tumor tissue overexpressed IL-6 in both a mouse animal model and human tissue. The mmu-miR-5112 is up-regulated in the adipocytes co-cultured with double-positive breast cancer cells. This miRNA targets and thus inhibits the expression of Cpeb1 and inhibitor of IL-6 [162].

The breast cancer cell lines, co-cultured with all types of adipose cells, show an overexpression of pro-inflammatory cytokines: IL-6, IL-8, IP-10, CCL2 and CCL5. This interaction does not affect the proliferation rate of breast cancer cells. However, in the case of co-culture of premature adipocytes with BC cells, this results in stimulation of stemness of cancer cells, mediated by miR-302f overexpression and its positive feedback with cMYC and SOX2 mRNA. Another thing to consider is that obese people have more predominance of pre-adipocytes in the adipose tissue [163].

## 5. Conclusions

The distribution of fat in the obese body influences the susceptibility of BC. A higher visceral adiposity leads to a greater risk of BC due to the systemic, as well as local imbalance at hormonal, inflammatory and non-coding RNA profile. The adiponectin-leptin-estrogen axis is altered, resulting in a decreased level of adiponectin, while leptin and estrogen levels are increased. The NFκB, JAK, STAT3, AKT signaling pathways are activated, as a consequence of this unbalance. The oxidative stress is greater in the case of obese people, because the adipose tissue secretes a greater quantity of pro-inflammatory cytokines, such as IL-1β, IL-6, IL-8, and TNFα, and exhibits the formation of inflammasome NLRP3 and NLRP4. The visceral fat in the case of obesity also releases an increased quantity of the BC oncomiRs - miR-23, and miR-155, miR-10b, miR-140, miR-302f, and a decreased quantity of the tumor suppressor miR-148b. Further in vitro studies of co-cultures of visceral adipocytes and breast cancer cells or in vivo studies of adipose tissue co-transplanted with breast cancer cells are needed. In addition, the epidemiological data related strictly to central obesity is still scarce and further analysis in this regard would also offer more valuable information. All of these will ultimately result in a more complete and informed understanding of the health risks associated with obesity in the general population.

## Figures and Tables

**Table 1:** Proinflammatory cytokines involved in tissue inflammation and Breast Cancer (BC) development.

Cytokine	Source of Secretion	Immune Significance	Role in BC	Ref.
IL-1β	Myeloid cells, Macrophages, T cells	- stimulates secretion of other pro-inflammatory mediators	- leads to poor prognostic - promotes tumor growth, and metastasis - induces invasiveness in TNBC (triple negative BC)	[54,55,56]
IL-6	T cells, Macrophages	- binds to IL6sR (soluble receptor) and stimulates STAT3 activation in cells that express gp130 - enhances inflammation - fights against infections	- leads to poor prognostic - increased levels in advanced breast tumor stages	[57,58]
IL-8	Macrophages, Epithelial cells, Smooth muscle cells, Endothelial cells	- has increased levels in inflammation - plays a key role in neutrophil degranulation	- increases metastatic potential of ER-, and ER+ BC cells - highly expressed in ER- BC - induces BC progression by stimulating invasion/metastasis	[59,60]
IL-17	Th17	- leads to inflammatory diseases such as Rheumatoid arthritis. - induces allergic responses - enhances inflammation by recruiting other proinflammatory cytokines	- induces overexpression of VEGF - promotes tumor cells survival - inhibits the antitumor immune response	[61,62]
IL-18	Macrophages	- together with IL-12, induces cell mediated immunity	- inhibits BC cell proliferation - induces inflammatory reactions - enhances BC migration - poor prognostic in TNBC	[56,63,64,65]
TNFα	Macrophages, NK cells, Neutrophils, Mast cells, Eosinophils	- relevant in inflammatory setup in BC cells - key proinflammatory cytokine	- enhances inflammatory process -mediates NFκB signaling pathway - regulates the immune cells - induces fever, and apoptosis - promotes BC migration	[66,67,68]

**Table 2:** List of microRNAs with differential expression in obese VAT versus normal VAT and in BC tissue versus normal tissue.

Name	Expression in Obese VAT	Expression in BC	Target Gene(s)	Biological Effect in BC	Ref.
miR-148b	↓	↓	*Dnmt3b, Itga5, Alcam*	proliferation, malignant transformation, pro-apoptosis, radio sensitivity, invasion, migration	[122,123,124,125,126]
miR-23	↑	↑	*Par2*	invasion, lung metastasis	[122,133,134,135,136]
miR-200	↑	↑	*E-Cadherin* *EPHA2*	invasion, migration, proliferation	[137,138,139,140]
miR-141	↑	↓			[137,141,142]
miR-429	↑	↓			[137,143]
miR-155	↑	↑	*Smad1/5, Casp3, Fadd, Rip1, Irak, Pka, Apaf-1, Foxo3a, Bcl-2, Socs1, Smad2, Hif, Pu.1,Mcsf, Tp53inp1*	Invasion, apoptosis, proliferation, angiogenesis, differentiation	[144,145,146]
miR-20b	↑	↑	Pten*	Cell growth and proliferation	[149,150]
miR-296	↑	↓	*hTERT*	Shorter telomeres, induce apoptosis	[150,151]
let-7f	↑	↑	*THBS1*	No effect on apoptosis or proliferation	[150,152]

* changes observed only at the protein level.

## Abbreviations

BC	Breast Cancer
TNBC	Triple Negative Breast Cancer
ER	Estrogen Receptor
PR	Progesteron Receptor
HER2	Human Epidermal Growth Factor Receptor 2
WAT	White Adipose Tissue
BMI	Body Mass Index
PR+	Progesterone Receptor Positive
VAT	Visceral Adipose Tissue
TAMS	Tumor Associated Macrophages
Tregs	Regulatory T Cells
Th2	Type 2 T Helper Cells
Bregs	Regulatory B Cells
CAF	Cancer Associated Fibroblasts
M1	Type 1 Macrophages
M2	Type 2 Macrophages
NK	Natural Killer Cells
INF γ	Interferon Gamma
TNFα	Tumor Necrosis Factor Alpha
IL	Interleukin
ROS	Reactive Oxygen Species
STAT3	Signal Transducer and Activator of Transcription 3
NRF 2	Nuclear Factor 2
AP1	Activator Protein 1
HIF 1α	Hypoxia-Inducible Factor 1 Alpha
PPAR	Peroxisome Proliferator-Activated Receptor
MMPs	Matrix Metalloproteinases
AKT	Protein Kinase B
NFκβ	Nuclear Factor Kappa-Light-Chain-Enhancer of Activated B Cells
TLR	Toll-Like Receptor
PAMPs	Pathogen-Associated Molecular Patterns
CRP	C Reactive Protein
LPS	Lipopolysaccharide
MCP 1	Monocyte Chemoattractant Protein 1
ICAM1	Intercellular Adhesion Molecule 1 (Cd54)
MIP	Macrophage Inflammatory Proteins
VEGF	Vascular Endothelial Growth Factor
ATC	Subcutaneous Stem Cells
NLRP3	Cryopyrin
KO	Knock-Out
WT	Wild Type
COX 2	Cyclooxygenase 2
PGE2	Prostaglandin E2
cAMP	Cyclic Amp
JNK	Jun N-Terminal Kinase
E2	Estradiol
PI3K	Phosphoinositide 3-Kinase
BRCA1	Breast Cancer Type 1 Susceptibility Protein
KEAP 1	Kelch-Like ECH-Associated Protein 1
ARE	Antioxidant Response Element
PP	Physagulide P
EGFR	Epidermal Growth Factor Receptor
MAPK	Mitogen Activated Protein Kinase
FGF	Fibroblast Growth Factor
EPHA 2	Ephrin Type-A Receptor 2
ATM	Adipose Tissue Macrophage

## References

[1] Shah R., Rosso K., Nathanson S.D. (2014). Pathogenesis, prevention, diagnosis and treatment of breast cancer. World J. Clin. Oncol..

[2] Jurj A., Braicu C., Pop L.-A., Tomuleasa C., Gherman C.D., Berindan-Neagoe I. (2017). The new era of nanotechnology, an alternative to change cancer treatment. Drug Des. Dev. Ther..

[3] Tomuleasa C., Braicu C., Irimie A., Craciun L., Berindan-Neagoe I. (2014). Nanopharmacology in translational hematology and oncology. Int. J. Nanomed..

[4] Braicu C., Catana C., Calin G.A., Berindan-Neagoe I. (2014). NCRNA combined therapy as future treatment option for cancer. Curr. Pharm. Des..

[5] Onitilo A.A., Engel J.M., Greenlee R.T., Mukesh B.N. (2009). Breast cancer subtypes based on ER/PR and Her2 expression: Comparison of clinicopathologic features and survival. Clin. Med. Res..

[6] Chiorean R., Braicu C., Berindan-Neagoe I. (2013). Another review on triple negative breast cancer. Are we on the right way towards the exit from the labyrinth?. Breast.

[7] Elks C.M., Francis J. (2010). Central adiposity, systemic inflammation, and the metabolic syndrome. Curr. Hypertens. Rep..

[8] Chen M.J., Wu W.Y., Yen A.M., Fann J.C., Chen S.L., Chiu S.Y., Chen H.H., Chiou S.T. (2016). Body mass index and breast cancer: Analysis of a nation-wide population-based prospective cohort study on 1 393 985 Taiwanese women. Int. J. Obes..

[9] Suzuki R., Orsini N., Saji S., Key T.J., Wolk A. (2009). Body weight and incidence of breast cancer defined by estrogen and progesterone receptor status—A meta-analysis. Int. J. Cancer.

[10] Munsell M.F., Sprague B.L., Berry D.A., Chisholm G., Trentham-Dietz A. (2014). Body mass index and breast cancer risk according to postmenopausal estrogen-progestin use and hormone receptor status. Epidemiol. Rev..

[11] Xia X., Chen W., Li J., Chen X., Rui R., Liu C., Sun Y., Liu L., Gong J., Yuan P. (2014). Body mass index and risk of breast cancer: A nonlinear dose-response meta-analysis of prospective studies. Sci. Rep..

[12] Harvie M., Hooper L., Howell A.H. (2003). Central obesity and breast cancer risk: A systematic review. Obes. Rev..

[13] Shieh Y., Scott C.G., Jensen M.R., Norman A.D., Bertrand K.A., Pankratz V.S., Brandt K.R., Visscher D.W., Shepherd J.A., Tamimi R.M. (2019). Body mass index, mammographic density, and breast cancer risk by estrogen receptor subtype. Breast Cancer Res. BCR.

[14] Pierobon M., Frankenfeld C.L. (2013). Obesity as a risk factor for triple-negative breast cancers: A systematic review and meta-analysis. Breast Cancer Res. Treat..

[15] Godinho-Mota J.C.M., Martins K.A., Vaz-Goncalves L., Mota J.F., Soares L.R., Freitas-Junior R. (2018). Visceral adiposity increases the risk of breast cancer: A case-control study. Nutr. Hosp..

[16] Wang F., Liu L., Cui S., Tian F., Fan Z., Geng C., Cao X., Yang Z., Wang X., Liang H. (2017). Distinct Effects of Body Mass Index and Waist/Hip Ratio on Risk of Breast Cancer by Joint Estrogen and Progestogen Receptor Status: Results from a Case-Control Study in Northern and Eastern China and Implications for Chemoprevention. Oncologist.

[17] Gu J.W., Young E., Patterson S.G., Makey K.L., Wells J., Huang M., Tucker K.B., Miele L. (2011). Postmenopausal obesity promotes tumor angiogenesis and breast cancer progression in mice. Cancer Biol. Ther..

[18] Liu C.R., Li Q., Hou C., Li H., Shuai P., Zhao M., Zhong X.R., Xu Z.P., Li J.Y. (2018). Changes in Body Mass Index, Leptin, and Leptin Receptor Polymorphisms and Breast Cancer Risk. DNA Cell Biol..

[19] Jung S.Y., Papp J.C., Sobel E.M., Yu H., Zhang Z.F. (2019). Breast cancer risk and insulin resistance: Post genome-wide gene-environment interaction study using a random survival forest. Cancer Res..

[20] Sanderson M., Lipworth L., Shrubsole M.J., Andersen S.W., Shu X.O., Zheng W., Hargreaves M.K., Blot W.J. (2019). Diabetes, obesity, and subsequent risk of postmenopausal breast cancer among white and black women in the Southern Community Cohort Study. Cancer Causes Control CCC.

[21] Martinez J.A., Chalasani P., Thomson C.A., Roe D., Altbach M., Galons J.-P., Stopeck A., Thompson P.A., Villa-Guillen D.E., Chow H.H.S. (2016). Phase II study of metformin for reduction of obesity-associated breast cancer risk: A randomized controlled trial protocol. BMC Cancer.

[22] Aldea M., Craciun L., Tomuleasa C., Berindan-Neagoe I., Kacso G., Florian I.S., Crivii C. (2014). Repositioning metformin in cancer: Genetics, drug targets, and new ways of delivery. Tumor Biol..

[23] Ando S., Gelsomino L., Panza S., Giordano C., Bonofiglio D., Barone I., Catalano S. (2019). Obesity, Leptin and Breast Cancer: Epidemiological Evidence and Proposed Mechanisms. Cancers.

[24] Noy R., Pollard J.W. (2014). Tumor-associated macrophages: From mechanisms to therapy. Immunity.

[25] Hadrup S., Donia M., Thor Straten P. (2013). Effector CD4 and CD8 T cells and their role in the tumor microenvironment. Cancer Microenviron..

[26] Schirrmacher V., Feuerer M., Beckhove P., Ahlert T., Umansky V. (2002). T cell memory, anergy and immunotherapy in breast cancer. J. Mammary Gland. Boil. Neoplas..

[27] Varn F.S., Mullins D.W., Arias-Pulido H., Fiering S., Cheng C. (2017). Adaptive immunity programmes in breast cancer. Immunology.

[28] He Y., Qian H., Liu Y., Duan L., Li Y., Shi G. (2014). The roles of regulatory B cells in cancer. J. Immunol. Res..

[29] Cannon B., Nedergaard J. (2004). Brown adipose tissue: Function and physiological significance. Physiol. Rev..

[30] Saely C.H., Geiger K., Drexel H. (2012). Brown versus white adipose tissue: A mini-review. Gerontology.

[31] Ibrahim M.M. (2010). Subcutaneous and visceral adipose tissue: Structural and functional differences. Obes. Rev..

[32] Donohoe C.L., Doyle S.L., Reynolds J.V. (2011). Visceral adiposity, insulin resistance and cancer risk. Diabetol. Metab. Syndr..

[33] Donninelli G., Del Corno M., Pierdominici M., Scazzocchio B., Vari R., Varano B., Pacella I., Piconese S., Barnaba V., D’Archivio M. (2017). Distinct Blood and Visceral Adipose Tissue Regulatory T Cell and Innate Lymphocyte Profiles Characterize Obesity and Colorectal Cancer. Front. Immunol..

[34] Piconese S., Valzasina B., Colombo M.P. (2008). OX40 triggering blocks suppression by regulatory T cells and facilitates tumor rejection. J. Exp. Med..

[35] Altintas M.M., Azad A., Nayer B., Contreras G., Zaias J., Faul C., Reiser J., Nayer A. (2011). Mast cells, macrophages, and crown-like structures distinguish subcutaneous from visceral fat in mice. J. Lipid Res..

[36] Zhou Y., Yu X., Chen H., Sjoberg S., Roux J., Zhang L., Ivoulsou A.H., Bensaid F., Liu C.L., Liu J. (2015). Leptin Deficiency Shifts Mast Cells toward Anti-Inflammatory Actions and Protects Mice from Obesity and Diabetes by Polarizing M2 Macrophages. Cell Metabolism.

[37] Ng M.F. (2010). The role of mast cells in wound healing. Int. Wound J..

[38] Mukai K., Tsai M., Saito H., Galli S.J. (2018). Mast cells as sources of cytokines, chemokines, and growth factors. Immunol. Rev..

[39] Bahr I., Goritz V., Doberstein H., Hiller G.G., Rosenstock P., Jahn J., Portner O., Berreis T., Mueller T., Spielmann J. (2017). Diet-Induced Obesity Is Associated with an Impaired NK Cell Function and an Increased Colon Cancer Incidence. J. Nutr. Metab..

[40] Conroy M.J., Fitzgerald V., Doyle S.L., Channon S., Useckaite Z., Gilmartin N., O’Farrelly C., Ravi N., Reynolds J.V., Lysaght J. (2016). The microenvironment of visceral adipose tissue and liver alter natural killer cell viability and function. J. Leukoc. Biol..

[41] Shoae-Hassani A., Behfar M., Mortazavi-Tabatabaei S.A., Ai J., Mohseni R., Hamidieh A.A. (2017). Natural Killer Cells from the Subcutaneous Adipose Tissue Underexpress the NKp30 and NKp44 in Obese Persons and Are Less Active against Major Histocompatibility Complex Class I Non-Expressing Neoplastic Cells. Front. Immunol..

[42] Moulin C.M., Rizzo L.V., Halpern A. (2008). Effect of surgery-induced weight loss on immune function. Expert Rev. Gastroenterol. Hepatol..

[43] Wouters K., Gaens K., Bijnen M., Verboven K., Jocken J., Wetzels S., Wijnands E., Hansen D., van Greevenbroek M., Duijvestijn A. (2017). Circulating classical monocytes are associated with CD11c(+) macrophages in human visceral adipose tissue. Sci. Rep..

[44] Tallerico R., Conti L., Lanzardo S., Sottile R., Garofalo C., Wagner A.K., Johansson M.H., Cristiani C.M., Karre K., Carbone E. (2017). NK cells control breast cancer and related cancer stem cell hematological spread. Oncoimmunology.

[45] Blaszczak A.M., Jalilvand A., Liu J., Wright V.P., Suzo A., Needleman B., Noria S., Lafuse W., Hsueh W.A., Bradley D. (2019). Human Visceral Adipose Tissue Macrophages Are Not Adequately Defined by Standard Methods of Characterization. J. Diabetes Res..

[46] Atri C., Guerfali F.Z., Laouini D. (2018). Role of Human Macrophage Polarization in Inflammation during Infectious Diseases. Int. J. Mol. Sci..

[47] Huda S.S., Jordan F., Bray J., Love G., Payne R., Sattar N., Freeman D.J. (2017). Visceral adipose tissue activated macrophage content and inflammatory adipokine secretion is higher in pre-eclampsia than in healthy pregnancys. Clin. Sci..

[48] Kralova Lesna I., Kralova A., Cejkova S., Fronek J., Petras M., Sekerkova A., Thieme F., Janousek L., Poledne R. (2016). Characterisation and comparison of adipose tissue macrophages from human subcutaneous, visceral and perivascular adipose tissue. J. Trans. Med..

[49] Fontana L., Eagon J.C., Trujillo M.E., Scherer P.E., Klein S. (2007). Visceral fat adipokine secretion is associated with systemic inflammation in obese humans. Diabetes.

[50] Williams C.B., Yeh E.S., Soloff A.C. (2016). Tumor-associated macrophages: Unwitting accomplices in breast cancer malignancy. NPJ Breast Cancer.

[51] Stoll B.A. (2002). Upper abdominal obesity, insulin resistance and breast cancer risk. Int. J. Obes. Relat. Metab. Disord..

[52] Koenen T.B., Stienstra R., van Tits L.J., Joosten L.A., van Velzen J.F., Hijmans A., Pol J.A., van der Vliet J.A., Netea M.G., Tack C.J. (2011). The inflammasome and caspase-1 activation: A new mechanism underlying increased inflammatory activity in human visceral adipose tissue. Endocrinology.

[53] Cancello R., Tordjman J., Poitou C., Guilhem G., Bouillot J.L., Hugol D., Coussieu C., Basdevant A., Bar Hen A., Bedossa P. (2006). Increased infiltration of macrophages in omental adipose tissue is associated with marked hepatic lesions in morbid human obesity. Diabetes.

[54] Reuter S., Gupta S.C., Chaturvedi M.M., Aggarwal B.B. (2010). Oxidative stress, inflammation, and cancer: How are they linked?. Free Radic. Biol. Med..

[55] Johar R., Sharma R., Kaur A., Mukherjee T.K. (2015). Role of Reactive Oxygen Species in Estrogen Dependant Breast Cancer Complication. Anticancer Agents Med. Chem..

[56] Madeddu C., Gramignano G., Floris C., Murenu G., Sollai G., Maccio A. (2014). Role of inflammation and oxidative stress in post-menopausal oestrogen-dependent breast cancer. J. Cell. Mol. Med..

[57] Teslow E.A., Mitrea C., Bao B., Mohammad R.M., Polin L.A., Dyson G., Purrington K.S., Bollig-Fischer A. (2019). Obesity-induced MBD2_v2 expression promotes tumor-initiating triple-negative breast cancer stem cells. Mol. Oncol..

[58] Bassols J., Ortega F.J., Moreno-Navarrete J.M., Peral B., Ricart W., Fernandez-Real J.M. (2009). Study of the proinflammatory role of human differentiated omental adipocytes. J. Cell. Biochem..

[59] Nagahashi M., Yamada A., Katsuta E., Aoyagi T., Huang W.C., Terracina K.P., Hait N.C., Allegood J.C., Tsuchida J., Yuza K. (2018). Targeting the SphK1/S1P/S1PR1 axis that links obesity, chronic inflammation and breast cancer metastasis. Cancer Res..

[60] Ritter A., Friemel A., Fornoff F., Adjan M., Solbach C., Yuan J., Louwen F. (2015). Characterization of adipose-derived stem cells from subcutaneous and visceral adipose tissues and their function in breast cancer cells. Oncotarget.

[61] Kantono M., Guo B. (2017). Inflammasomes and Cancer: The Dynamic Role of the Inflammasome in Tumor Development. Front. Immunol..

[62] Guo B., Fu S., Zhang J., Liu B., Li Z. (2016). Targeting inflammasome/IL-1 pathways for cancer immunotherapy. Sci. Rep..

[63] Kolb R., Phan L., Borcherding N., Liu Y., Yuan F., Janowski A.M., Xie Q., Markan K.R., Li W., Potthoff M.J. (2016). Obesity-associated NLRC4 inflammasome activation drives breast cancer progression. Nat. Commun..

[64] Jeon M., Han J., Nam S.J., Lee J.E., Kim S. (2016). Elevated IL-1β expression induces invasiveness of triple negative breast cancer cells and is suppressed by zerumbone. Chem. Biol. Interact..

[65] Lin S., Gan Z., Han K., Yao Y., Min D. (2015). Interleukin-6 as a prognostic marker for breast cancer: A meta-analysis. Tumori.

[66] Heinrich P.C., Behrmann I., Haan S., Hermanns H.M., Muller-Newen G., Schaper F. (2003). Principles of interleukin (IL)-6-type cytokine signalling and its regulation. Biochem. J..

[67] Sullivan N.J. (2011). Interleukin-6 in Breast Tumor Microenvironment, Breast Cancer—Focusing Tumor Microenvironment, Stem Cells and Metastasis.

[68] Harada A., Sekido N., Akahoshi T., Wada T., Mukaida N., Matsushima K. (1994). Essential involvement of interleukin-8 (IL-8) in acute inflammation. J. Leukoc. Biol..

[69] Todorovic-Rakovic N., Milovanovic J. (2013). Interleukin-8 in breast cancer progression. J. Interf. Cytokine Res..

[70] Kawaguchi M., Kokubu F., Fujita J., Huang S.K., Hizawa N. (2009). Role of interleukin-17F in asthma. Inflamm. Allergy Drug Targets.

[71] Welte T., Zhang X.H. (2015). Interleukin-17 Could Promote Breast Cancer Progression at Several Stages of the Disease. Mediat. Inflamm..

[72] Nicolini A., Carpi A., Rossi G. (2006). Cytokines in breast cancer. Cytokine Growth Factor Rev..

[73] Huang H.Y., Yu H.T., Chan S.H., Lee C.L., Wang H.S., Soong Y.K. (2010). Eutopic endometrial interleukin-18 system mRNA and protein expression at the level of endometrial-myometrial interface in adenomyosis patients. Fertil. Steril..

[74] Park I.H., Yang H.N., Lee K.J., Kim T.S., Lee E.S., Jung S.Y., Kwon Y., Kong S.Y. (2017). Tumor-derived IL-18 induces PD-1 expression on immunosuppressive NK cells in triple-negative breast cancer. Oncotarget.

[75] Lu L., Shi W., Deshmukh R.R., Long J., Cheng X., Ji W., Zeng G., Chen X., Zhang Y., Dou Q.P. (2014). Tumor necrosis factor-alpha sensitizes breast cancer cells to natural products with proteasome-inhibitory activity leading to apoptosis. PLoS ONE.

[76] Locksley R.M., Killeen N., Lenardo M.J. (2001). The TNF and TNF receptor superfamilies: Integrating mammalian biology. Cell.

[77] Gao Y., Yang Y., Yuan F., Huang J., Xu W., Mao B., Yuan Z., Bi W. (2017). TNFalpha-YAP/p65-HK2 axis mediates breast cancer cell migration. Oncogenesis.

[78] Sadashiv, Tiwari S., Paul B.N., Kumar S., Chandra A., Dhananjai S., Negi M.P. (2012). Resistin gene expression in visceral adipose tissue of postmenopausal women and its association with insulin resistance. Womens Health.

[79] Curat C.A., Wegner V., Sengenes C., Miranville A., Tonus C., Busse R., Bouloumie A. (2006). Macrophages in human visceral adipose tissue: Increased accumulation in obesity and a source of resistin and visfatin. Diabetologia.

[80] Coelho M., Oliveira T., Fernandes R. (2013). Biochemistry of adipose tissue: An endocrine organ. Arch. Med. Sci..

[81] Yip I., Go V.L., Hershman J.M., Wang H.J., Elashoff R., DeShields S., Liu Y., Heber D. (2001). Insulin-leptin-visceral fat relation during weight loss. Pancreas.

[82] Niu J., Jiang L., Guo W., Shao L., Liu Y., Wang L. (2013). The Association between Leptin Level and Breast Cancer: A Meta-Analysis. PLoS ONE.

[83] Newman G., Gonzalez-Perez R.R. (2014). Leptin-cytokine crosstalk in breast cancer. Mol. Cell. Endocrinol..

[84] Haque I., Ghosh A., Acup S., Banerjee S., Dhar K., Ray A., Sarkar S., Kambhampati S., Banerjee S.K. (2018). Leptin-induced ER-alpha-positive breast cancer cell viability and migration is mediated by suppressing CCN5-signaling via activating JAK/AKT/STAT-pathway. BMC Cancer.

[85] Raut P.K., Choi D.Y., Kim S.H., Hong J.T., Kwon T.K., Jeong J.H., Park P.H. (2017). Estrogen receptor signaling mediates leptin-induced growth of breast cancer cells via autophagy induction. Oncotarget.

[86] Mocino-Rodriguez M.D., Santillan-Benitez J.G., Dozal-Dominguez D.S., Hernandez-Navarro M.D., Flores-Merino M.V., Sandoval-Cabrera A., Garcia Vazquez F.J. (2017). Expression of AdipoR1 and AdipoR2 Receptors as Leptin-Breast Cancer Regulation Mechanisms. Dis. Mark..

[87] Gonzalez-Perez R.R., Lanier V., Newman G. (2013). Leptin’s Pro-Angiogenic Signature in Breast Cancer. Cancers.

[88] Rene Gonzalez R., Watters A., Xu Y., Singh U.P., Mann D.R., Rueda B.R., Penichet M.L. (2009). Leptin-signaling inhibition results in efficient anti-tumor activity in estrogen receptor positive or negative breast cancer. Breast Cancer Res. BCR.

[89] Pena-Cano M.I., Saucedo R., Morales-Avila E., Valencia J., Zavala-Moha J.A., Lopez A. (2019). Deregulated microRNAs and Adiponectin in Postmenopausal Women with Breast Cancer. Gynecol. Obstet. Investig..

[90] Yu Z., Tang S., Ma H., Duan H., Zeng Y. (2019). Association of serum adiponectin with breast cancer: A meta-analysis of 27 case-control studies. Medicine.

[91] Macías-Gómez N.M., Hernández-Terrones M.C., Ramírez-Guerrero A.A., Leal-Ugarte E., Gutiérrez-Angulo M., Peregrina-Sandoval J. (2019). ADIPOQ rs2241766 SNP as protective marker against DIBC development in Mexican population. PLoS ONE.

[92] Chung S.J., Nagaraju G.P., Nagalingam A., Muniraj N., Kuppusamy P., Walker A., Woo J., Gyorffy B., Gabrielson E., Saxena N.K. (2017). ADIPOQ/adiponectin induces cytotoxic autophagy in breast cancer cells through STK11/LKB1-mediated activation of the AMPK-ULK1 axis. Autophagy.

[93] Mauro L., Naimo G.D., Gelsomino L., Malivindi R., Bruno L., Pellegrino M., Tarallo R., Memoli D., Weisz A., Panno M.L. (2018). Uncoupling effects of estrogen receptor alpha on LKB1/AMPK interaction upon adiponectin exposure in breast cancer. FASEB J. Off. Publ. Fed. Am. Soc. Exp. Biol..

[94] Fruhbeck G. (2006). Intracellular signalling pathways activated by leptin. Biochem. J..

[95] Vansaun M.N. (2013). Molecular pathways: Adiponectin and leptin signaling in cancer. Clin. Cancer Res. Off. J. Am. Assoc. Cancer Res..

[96] Nanjappa V., Raju R., Muthusamy B., Sharma J., Thomas J.K., Nidhina P.A.H., Harsha H.C., Pandey A., Anilkumar G., Prasad T.S.K. (2011). A comprehensive curated reaction map of leptin signaling pathway. J. Proteom. Bioinform..

[97] Van Kruijsdijk R.C., van der Wall E., Visseren F.L. (2009). Obesity and cancer: The role of dysfunctional adipose tissue. Cancer Epidemiol. Biomark. Prev. Publ. Am. Assoc. Cancer Res. Cospons. Am. Soc. Prev. Oncol..

[98] Nalabolu M.R., Palasamudram K., Jamil K. (2014). Adiponectin and leptin molecular actions and clinical significance in breast cancer. Int. J. Hematol. Oncol. Stem Cell Res..

[99] Ruan H., Dong L.Q. (2016). Adiponectin signaling and function in insulin target tissues. J. Mol. Cell Biol..

[100] Kim H.G., Jin S.W., Kim Y.A., Khanal T., Lee G.H., Kim S.J., Rhee S.D., Chung Y.C., Hwang Y.J., Jeong T.C. (2017). Leptin induces CREB-dependent aromatase activation through COX-2 expression in breast cancer cells. Food Chem. Toxicol..

[101] Liao W., Yu C., Wen J., Jia W., Li G., Ke Y., Zhao S., Campell W. (2009). Adiponectin induces interleukin-6 production and activates STAT3 in adult mouse cardiac fibroblasts. Biol. Cell.

[102] Magoffin D.A., Weitsman S.R., Aagarwal S.K., Jakimiuk A.J. (1999). Leptin regulation of aromatase activity in adipose stromal cells from regularly cycling women. Ginekol. Pol..

[103] Catalano S., Marsico S., Giordano C., Mauro L., Rizza P., Panno M.L., Ando S. (2003). Leptin enhances, via AP-1, expression of aromatase in the MCF-7 cell line. J. Biol. Chem..

[104] Tekmal R.R., Kirma N., Gill K., Fowler K. (1999). Aromatase overexpression and breast hyperplasia, an in vivo model—Continued overexpression of aromatase is sufficient to maintain hyperplasia without circulating estrogens, and aromatase inhibitors abrogate these preneoplastic changes in mammary glands. Endocr. Relat. Cancer.

[105] Cleary M.P., Grossmann M.E. (2009). Minireview: Obesity and breast cancer: The estrogen connection. Endocrinology.

[106] Hetemaki N., Savolainen-Peltonen H., Tikkanen M.J., Wang F., Paatela H., Hamalainen E., Turpeinen U., Haanpaa M., Vihma V., Mikkola T.S. (2017). Estrogen Metabolism in Abdominal Subcutaneous and Visceral Adipose Tissue in Postmenopausal Women. J. Clin. Endocrinol. Metab..

[107] Perez-Hernandez A.I., Catalan V., Gomez-Ambrosi J., Rodriguez A., Fruhbeck G. (2014). Mechanisms linking excess adiposity and carcinogenesis promotion. Front. Endocrinol..

[108] Liang J., Shang Y. (2013). Estrogen and cancer. Annu. Rev. Physiol..

[109] Gorrini C., Gang B.P., Bassi C., Wakeham A., Baniasadi S.P., Hao Z., Li W.Y., Cescon D.W., Li Y.T., Molyneux S. (2014). Estrogen controls the survival of BRCA1-deficient cells via a PI3K-NRF2-regulated pathway. Proc. Natl. Acad. Sci. USA.

[110] Jeffery E., Wing A., Holtrup B., Sebo Z., Kaplan J.L., Saavedra-Pena R., Church C.D., Colman L., Berry R., Rodeheffer M.S. (2016). The Adipose Tissue Microenvironment Regulates Depot-Specific Adipogenesis in Obesity. Cell Metab..

[111] Kaaks R., Rinaldi S., Key T.J., Berrino F., Peeters P.H., Biessy C., Dossus L., Lukanova A., Bingham S., Khaw K.T. (2005). Postmenopausal serum androgens, oestrogens and breast cancer risk: The European prospective investigation into cancer and nutrition. Endocr. Relat. Cancer.

[112] Key T.J., Appleby P.N., Reeves G.K., Roddam A., Dorgan J.F., Longcope C., Stanczyk F.Z., Stephenson H.E., Falk R.T., Miller R. (2003). Body mass index, serum sex hormones, and breast cancer risk in postmenopausal women. J. Natl. Cancer Instig..

[113] Yager J.D., Davidson N.E. (2006). Estrogen carcinogenesis in breast cancer. New Engl. J. Med..

[114] Fuhrman B.J., Schairer C., Gail M.H., Boyd-Morin J., Xu X., Sue L.Y., Buys S.S., Isaacs C., Keefer L.K., Veenstra T.D. (2012). Estrogen metabolism and risk of breast cancer in postmenopausal women. J. Natl. Cancer Inst..

[115] Stender J.D., Frasor J., Komm B., Chang K.C., Kraus W.L., Katzenellenbogen B.S. (2007). Estrogen-regulated gene networks in human breast cancer cells: Involvement of E2F1 in the regulation of cell proliferation. Mol. Endocrinol..

[116] Ruan X., Seeger H., Wallwiener D., Huober J., Mueck A.O. (2015). The ratio of the estradiol metabolites 2-hydroxyestrone (2-OHE1) and 16alpha-hydroxyestrone (16-OHE1) may predict breast cancer risk in postmenopausal but not in premenopausal women: Two case-control studies. Arch. Gynecol. Obstet..

[117] Tripathi K., Mani C., Somasagara R.R., Clark D.W., Ananthapur V., Vinaya K., Palle K. (2017). Detection and evaluation of estrogen DNA-adducts and their carcinogenic effects in cultured human cells using biotinylated estradiol. Mol. Carcinogen..

[118] Bradlow H.L., Telang N.T., Sepkovic D.W., Osborne M.P. (1996). 2-hydroxyestrone: The ‘good’ estrogen. J. Endocrinol..

[119] Obi N., Vrieling A., Heinz J., Chang-Claude J. (2011). Estrogen metabolite ratio: Is the 2-hydroxyestrone to 16alpha-hydroxyestrone ratio predictive for breast cancer?. Int. J. Womens Health.

[120] Yue W., Yager J.D., Wang J.P., Jupe E.R., Santen R.J. (2013). Estrogen receptor-dependent and independent mechanisms of breast cancer carcinogenesis. Steroids.

[121] Berindan-Neagoe I., Calin G.A. (2014). Molecular pathways: MicroRNAs, cancer cells, and microenvironment. Clin. Cancer Res. Off. J. Am. Assoc. Cancer Res..

[122] Ferrante S.C., Nadler E.P., Pillai D.K., Hubal M.J., Wang Z., Wang J.M., Gordish-Dressman H., Koeck E., Sevilla S., Wiles A.A. (2015). Adipocyte-derived exosomal miRNAs: A novel mechanism for obesity-related disease. Pediatric Res..

[123] Sandhu R., Rivenbark A.G., Coleman W.B. (2012). Loss of post-transcriptional regulation of DNMT3b by microRNAs: A possible molecular mechanism for the hypermethylation defect observed in a subset of breast cancer cell lines. Int. J. Oncol..

[124] Roll J.D., Rivenbark A.G., Jones W.D., Coleman W.B. (2008). DNMT3b overexpression contributes to a hypermethylator phenotype in human breast cancer cell lines. Mol. Cancer.

[125] Cimino D., De Pitta C., Orso F., Zampini M., Casara S., Penna E., Quaglino E., Forni M., Damasco C., Pinatel E. (2013). miR148b is a major coordinator of breast cancer progression in a relapse-associated microRNA signature by targeting ITGA5, ROCK1, PIK3CA, NRAS, and CSF1. FASEB J. Off. Publ. Fed. of Am. Soc. Exp. Biol..

[126] Orso F., Quirico L., Virga F., Penna E., Dettori D., Cimino D., Coppo R., Grassi E., Elia A.R., Brusa D. (2016). miR-214 and miR-148b Targeting Inhibits Dissemination of Melanoma and Breast Cancer. Cancer Res..

[127] Lai Y., Chen Y., Lin Y., Ye L. (2018). Down-regulation of LncRNA CCAT1 enhances radiosensitivity via regulating miR-148b in breast cancer. Cell. Biol. Int..

[128] Cuk K., Zucknick M., Madhavan D., Schott S., Golatta M., Heil J., Marme F., Turchinovich A., Sinn P., Sohn C. (2013). Plasma microRNA panel for minimally invasive detection of breast cancer. PLoS ONE.

[129] Cuk K., Zucknick M., Heil J., Madhavan D., Schott S., Turchinovich A., Arlt D., Rath M., Sohn C., Benner A. (2013). Circulating microRNAs in plasma as early detection markers for breast cancer. Int. J. Cancer.

[130] Shen J., Hu Q., Schrauder M., Yan L., Wang D., Medico L., Guo Y., Yao S., Zhu Q., Liu B. (2014). Circulating miR-148b and miR-133a as biomarkers for breast cancer detection. Oncotarget.

[131] Li J.Y., Jia S., Zhang W.H., Zhang Y., Kang Y., Li P.S. (2013). Differential distribution of microRNAs in breast cancer grouped by clinicopathological subtypes. Asian Pac. J. Cancer Prev..

[132] Do Canto L.M., Marian C., Willey S., Sidawy M., Da Cunha P.A., Rone J.D., Li X., Gusev Y., Haddad B.R. (2016). MicroRNA analysis of breast ductal fluid in breast cancer patients. Int. J. Oncol..

[133] Schultz D.J., Muluhngwi P., Alizadeh-Rad N., Green M.A., Rouchka E.C., Waigel S.J., Klinge C.M. (2017). Genome-wide miRNA response to anacardic acid in breast cancer cells. PLoS ONE.

[134] Lewinska A., Adamczyk-Grochala J., Deregowska A., Wnuk M. (2017). Sulforaphane-Induced Cell Cycle Arrest and Senescence are accompanied by DNA Hypomethylation and Changes in microRNA Profile in Breast Cancer Cells. Theranostics.

[135] Pellegrino L., Stebbing J., Braga V.M., Frampton A.E., Jacob J., Buluwela L., Jiao L.R., Periyasamy M., Madsen C.D., Caley M.P. (2013). miR-23b regulates cytoskeletal remodeling, motility and metastasis by directly targeting multiple transcripts. Nucl. Acids Res..

[136] Ell B., Qiu Q., Wei Y., Mercatali L., Ibrahim T., Amadori D., Kang Y. (2014). The microRNA-23b/27b/24 cluster promotes breast cancer lung metastasis by targeting metastasis-suppressive gene prosaposin. J. Biol. Chem..

[137] Adi N., Adi J., Cesar L., Kurlansky P., Agatston A., Webster K.A. (2015). Role of Micro RNA-205 in Promoting Visceral Adiposity of NZ10 Mice with Polygenic Susceptibility for Type 2 Diabetes. J. Diabetes Metab..

[138] Feng X., Wang Z., Fillmore R., Xi Y. (2014). MiR-200, a new star miRNA in human cancer. Cancer Lett..

[139] Tsouko E., Wang J., Frigo D.E., Aydogdu E., Williams C. (2015). miR-200a inhibits migration of triple-negative breast cancer cells through direct repression of the EPHA2 oncogene. Carcinogenesis.

[140] Yao J., Xu F., Zhang D., Yi W., Chen X., Chen G., Zhou E. (2018). TP73-AS1 promotes breast cancer cell proliferation through miR-200a-mediated TFAM inhibition. J. Cell. Biochem..

[141] Li P., Xu T., Zhou X., Liao L., Pang G., Luo W., Han L., Zhang J., Luo X., Xie X. (2017). Downregulation of miRNA-141 in breast cancer cells is associated with cell migration and invasion: Involvement of ANP32E targeting. Cancer Med..

[142] Abedi N., Mohammadi-Yeganeh S., Koochaki A., Karami F., Paryan M. (2015). miR-141 as potential suppressor of beta-catenin in breast cancer. Tumour Biol. J. Int. Soc. Oncodev. Biol. Med..

[143] Ye Z.B., Ma G., Zhao Y.H., Xiao Y., Zhan Y., Jing C., Gao K., Liu Z.H., Yu S.J. (2015). miR-429 inhibits migration and invasion of breast cancer cells in vitro. Int. J. Oncol..

[144] Ying W., Riopel M., Bandyopadhyay G., Dong Y., Birmingham A., Seo J.B., Ofrecio J.M., Wollam J., Hernandez-Carretero A., Fu W. (2017). Adipose Tissue Macrophage-Derived Exosomal miRNAs Can Modulate In Vivo and In Vitro Insulin Sensitivity. Cell.

[145] Mattiske S., Suetani R.J., Neilsen P.M., Callen D.F. (2012). The oncogenic role of miR-155 in breast cancer. Cancer Epidemiol. Biomark. Prev. Publ. Am. Assoc. Cancer Res. Cospons. Am. Soc. Prev. Oncol..

[146] Zhang C.M., Zhao J., Deng H.Y. (2013). MiR-155 promotes proliferation of human breast cancer MCF-7 cells through targeting tumor protein 53-induced nuclear protein 1. J. Biomed. Sci..

[147] Cojocneanu Petric R., Braicu C., Raduly L., Zanoaga O., Dragos N., Monroig P., Dumitrascu D., Berindan-Neagoe I. (2015). Phytochemicals modulate carcinogenic signaling pathways in breast and hormone-related cancers. OncoTargets Ther..

[148] Braicu C., Mehterov N., Vladimirov B., Sarafian V., Nabavi S.M., Atanasov A.G., Berindan-Neagoe I. (2017). Nutrigenomics in cancer: Revisiting the effects of natural compounds. Semin. Cancer Biol..

[149] Zhou W., Shi G., Zhang Q., Wu Q., Li B., Zhang Z. (2014). MicroRNA-20b promotes cell growth of breast cancer cells partly via targeting phosphatase and tensin homologue (PTEN). Cell Biosci..

[150] Gentile A.M., Lhamyani S., Coin-Araguez L., Clemente-Postigo M., Oliva Olivera W., Romero-Zerbo S.Y., Garcia-Serrano S., Garcia-Escobar E., Zayed H., Doblado E. (2019). miR-20b, miR-296, and Let-7f Expression in Human Adipose Tissue is Related to Obesity and Type 2 Diabetes. Obesity.

[151] Dinami R., Buemi V., Sestito R., Zappone A., Ciani Y., Mano M., Petti E., Sacconi A., Blandino G., Giacca M. (2017). Epigenetic silencing of miR-296 and miR-512 ensures hTERT dependent apoptosis protection and telomere maintenance in basal-type breast cancer cells. Oncotarget.

[152] Tao W.-Y., Liang X.-S., Liu Y., Wang C.-Y., Pang D. (2015). Decrease of let-7f in low-dose metronomic Paclitaxel chemotherapy contributed to upregulation of thrombospondin-1 in breast cancer. Int. J. Biol. Sci..

[153] Robado de Lope L., Alcíbar O.L., Amor López A., Hergueta-Redondo M., Peinado H. (2018). Tumour-adipose tissue crosstalk: Fuelling tumour metastasis by extracellular vesicles. Philos. Trans. R. Soc. Lond. B Biol. Sci..

[154] Ji C., Guo X. (2019). The clinical potential of circulating microRNAs in obesity. Nat. Rev. Endocrinol..

[155] Groza M., Zimta A.A., Irimie A., Achimas-Cadariu P., Cenariu D., Stanta G., Berindan-Neagoe I. (2019). Recent advancements in the study of breast cancer exosomes as mediators of intratumoral communication. J. Cell. Physiol..

[156] Gernapudi R., Yao Y., Zhang Y., Wolfson B., Roy S., Duru N., Eades G., Yang P., Zhou Q. (2015). Targeting exosomes from preadipocytes inhibits preadipocyte to cancer stem cell signaling in early-stage breast cancer. Breast Cancer Res. Treat..

[157] Lin R., Wang S., Zhao R.C. (2013). Exosomes from human adipose-derived mesenchymal stem cells promote migration through Wnt signaling pathway in a breast cancer cell model. Mol. Cell. Biochem..

[158] Wang S., Su X., Xu M., Xiao X., Li X., Li H., Keating A., Zhao R. (2019). Exosomes secreted by mesenchymal stromal/stem cell-derived adipocytes promote breast cancer cell growth via activation of Hippo signaling pathway. Stem Cell Res. Ther..

[159] Thomou T., Mori M.A., Dreyfuss J.M., Konishi M., Sakaguchi M., Wolfrum C., Rao T.N., Winnay J.N., Garcia-Martin R., Grinspoon S.K. (2017). Adipose-derived circulating miRNAs regulate gene expression in other tissues. Nature.

[160] Wu Q., Sun S., Li Z., Yang Q., Li B., Zhu S., Wang L., Wu J., Yuan J., Wang C. (2019). Breast cancer-released exosomes trigger cancer-associated cachexia to promote tumor progression. Adipocyte.

[161] Meerson A., Eliraz Y., Yehuda H., Knight B., Crundwell M., Ferguson D., Lee B.P., Harries L.W. (2019). Obesity impacts the regulation of miR-10b and its targets in primary breast tumors. BMC Cancer.

[162] Lee J., Suk Ryu H., Sil Hong B., Lee H.-B., Lee M., Ae Park I., Kim J., Han W., Noh D.-Y., Moon H.-G. (2017). Transition into inflammatory cancer-associated adipocytes in breast cancer microenvironment requires microRNA regulatory mechanism. PLoS ONE.

[163] Picon-Ruiz M., Pan C., Drews-Elger K., Jang K., Besser A.H., Zhao D., Morata-Tarifa C., Kim M., Ince T.A., Azzam D.J. (2016). Interactions between Adipocytes and Breast Cancer Cells Stimulate Cytokine Production and Drive Src/Sox2/miR-302b–Mediated Malignant Progression. Cancer Res..

